# Dental resins used in 3D printing technologies release ovo-toxic leachates

**DOI:** 10.1016/j.chemosphere.2020.129003

**Published:** 2021-05

**Authors:** Hunter B. Rogers, Luhan T. Zhou, Atsuko Kusuhara, Emily Zaniker, Saman Shafaie, Benjamin C. Owen, Francesca E. Duncan, Teresa K. Woodruff

**Affiliations:** a Department of Obstetrics and Gynecology, Northwestern University, Chicago, IL, 60611, USA; b Integrated Molecular Structure Education and Research Center (IMSERC), Northwestern University, Evanston, IL, 60208, USA

**Keywords:** Reproduction, Toxicity, Oocytes, Dental, Leachate, Light stabilizer, 3D-printing

## Abstract

We recently engineered the first female reproductive tract on a chip (EVATAR), to enable sex-based ex vivo research. To increase the scalability and accessibility of EVATAR, we turned to 3D printing (3DP) technologies, selecting two biocompatible 3DP resins, Dental SG (DSG) and Dental LT (DLT) to generate 3DP microphysiologic platforms. Due to the known sensitivity of reproductive cells to leachable compounds, we first screened for toxicity of these biomaterials using an in vitro mammalian oocyte maturation assay. Culture of mouse oocytes in 3DP plates using conventionally treated DSG resin resulted in rapid oocyte degeneration. Oxygen plasma treatment of the surface of printed DSG resin prevented this degeneration, and the majority of the resulting oocytes progressed through meiosis in vitro. However, 57.0% ± 37.2% of the cells cultured in the DSG resin plates exhibited abnormal chromosome morphology compared to 19.4% ± 17.3% of controls cultured in polystyrene. All tested DLT resin conditions, including plasma treatment, resulted in complete and rapid oocyte degeneration. To identify the ovo-toxic component of DLT, we analyzed DLT leachate using mass spectroscopy. We identified Tinuvin 292, a commercial light stabilizer, as a major component of the DLT leachate, which resulted in a dose-dependent disruption of meiotic progression and increase in chromosomal abnormalities with oocyte exposure, showing significant ovo-toxicity in mammals. Severe reproductive toxicity induced by in vitro exposure to these 3D-printed resins highlights potential risks of deploying insufficiently characterized materials for biomedical applications and underscores the need for more rigorous evaluation and designation of biocompatible materials.

## Highlights

• Exposure to dental resins commonly used for 3D printing technology causes mammalian oocyte toxicity in vitro .
• Dental LT resin exposure results in more severe oocyte toxicity compared to Dental SG.
• The commercial light stabilizer Tinuvin 292 leaches from Dental LT resin.
• Exposure to Tinuvin 292 mimics the oocyte toxicity observed with dental resins.
• Our findings underscore the need for more rigorous evaluation and designation of biocompatible materials.

## 1 Introduction

Additive manufacturing technologies, also known as 3D printing (3DP), enable the production of common or custom parts in a time- and cost-efficient manner which has catalyzed their uptake in new technologies and have redefined the prototyping processes across industries, such as construction, consumer goods, and aerospace (Bogers et al., 2016; Tay et al., 2017; Joshi and Sheikh, 2015). The recent development of biocompatible resins has led to rapid adoption of 3DP in the biomedical sciences for applications such as anatomical and surgical models, medical devices, and tissue engineered constructs (Ventola, 2014; Shafiee and Atala, 2016; Gargus et al., 2020). Increased affordability of 3DP systems and 3D model sharing services, such as the NIH 3D Print Exchange, have significantly lowered the barriers-to-entry for scientists and clinicians, which has created a new field of biomedical 3DP. These recent developments led our research group to use 3DP technology to engineer next-generation fluidic culture systems to create the first ex vivo model of the female reproductive tract (Xiao et al., 2017). This culture system, called EVATAR, enables the long-term culture of multiple tissues from organs of the female reproductive tract, including the ovary, fallopian tube, endometrium, and cervix, in a manner that recapitulates an idealized 28-day hormone profile of the human menstrual cycle. This system has the potential to advance basic biological research as well as drug discovery and reproductive toxicity screening using human cells well beyond traditional two-dimensional, static culture techniques. However, given the complexity of the EVATAR system, high financial and technical barriers have prevented accessibility to the broader research community. Therefore, there is a significant need to engineer a system with similar functionality but with greater accessibility as potentially afforded by 3DP technology.

However, a challenge to the translation of 3DP products into the field of biomedicine is the potential cytotoxicity of commonly used resins. Although biocompatibility is often defined as “the ability of a material to perform with an appropriate host response in a specific application,” this term and definition is still a topic of on-going debate (Williams, 1986, 2003; Ratner, 2016; Ghasemi-Mobarakeh et al., 2019). In practice, the term “biocompatible” is often used without specific context, and current approaches to standardization of testing for “biocompatibility”, such as ISO standards, have shortcomings. Indeed, despite being labeled “biocompatible,” leachates from several conventional plastics and current in-use medical devices can have a significant impact on public health and the environment (Pant et al., 2001; Teuten et al., 2009; Rustagi et al., 2011; Lee et al., 2015). While the release of cytotoxic plasticizers and other plastic additives, such as bisphenol A (BPA), from plastics manufactured using traditional methods is well known, there is a dearth of knowledge on the potential cytotoxicity of emerging 3DP products either in vivo in the case of implants or other medical devices, or ex vivo in the case of the cell culture devices our team are developing.

Therefore, to investigate the feasibility of utilizing 3DP materials to engineer devices to support the long-term culture of reproductive cells and tissues we analyzed two resins, Dental SG (DSG) and Dental LT Clear (DLT). These two 3DP resins were selected based on their ISO-certification as biocompatible and their current commercial use in medical applications, namely oral surgical guides and retainers. To screen for potential reproductive toxicity, we used an in vitro maturation (IVM) assay using mouse oocytes. The female gamete, or oocyte, has the developmental potential to give rise to the next generation and is sensitive to perturbations, including environmental, metabolic, or age-related (Ratchford et al., 2008; Wang et al., 2009; Wang and Moley, 2010; Duncan et al., 2012; Krisher, 2013; Pohlmeier et al., 2014). Meiotic maturation - or the process by which prophase I-arrested oocytes resume meiosis, complete meiosis I, and arrest at the metaphase II-arrested stage - is a key biological process required to ultimately generate gametes that can undergo normal fertilization and give rise to euploid offspring. Meiotic maturation represents a critical window of susceptibility because disruption of this process can result in chromosome segregation errors, leading to infertility, miscarriages, and birth defects. In fact, the reproductive toxicity of BPA was first identified through the detrimental effects of plastic leachates on mouse oocyte meiosis (Koehler et al., 2003; Hunt et al., 2003, 2012; Peretz et al., 2014). This knowledge has been accompanied by a wide body of literature demonstrating the sensitivity of the mammalian oocyte and the broader reproductive system to replacement bisphenols, pthalates, and other endocrine disrupting chemicals (Sartain and Hunt, 2016; Chiang et al., 2017; Horan et al., 2018; Vandenberg et al., 2019; Segal and Giudice, 2019; Brehm and Flaws 2019). Meiotic maturation occurs physiologically at the time of ovulation in response to the luteinizing hormone surge but can also be induced in vitro by removing the oocyte from the follicle. Thus, the oocyte is a highly responsive mammalian cell model, and the biological pathway of meiotic maturation is a sensitive and powerful screening assay for potential reproductive toxicity due to exposure to materials and compounds of interest.

Here we show evidence that DSG and DLT resins are toxic to the mammalian oocyte upon in vitro exposure, underscoring the importance of additional studies to determine their safety for their intended use in dental applications. Further, we identified Tinuvin 292, a hindered amine light stabilizer, as the leachate responsible for DLT’s reproductive toxicity and rapid degeneration of oocytes. Tinuvin 292 is a plastic additive used in a broad range of commercial products and, therefore, the risk of exposure extends well beyond the 3DP materials in this study. We did not set out to prove this, rather we made this discovery while using the resins to create new culture tools for reproductive tissues. Our studies raise a biological red flag, providing evidence that certification of biocompatible materials should require rigorous reproductive toxicity testing in all cases due to the potential of leaching *in vivo*. Moreover, the wide use of plastic additives such as Tinuvin 292 in commercial products should be further investigated to determine the potential risk due to exposure of materials containing these additives.

## 2 Materials and methods

### 2.1 3D printing with dental SG and dental LT

Computer-aided design (CAD) files were created using SketchUp (Trimble, Inc., United States) and imported to PreForm software (Formlabs, United States) for print preparation. Models were printed using Form 2 SLA printers (Formlabs, United States) using either Dental SG (FLDGOR01, Lot Nos. XN232N05, XK244N01, XK242N01, XK25N01, XH084N05) or Dental LT Clear (FLDLCL01, Lot Nos. XK484N02, XH043N02, XK292N02) resins (Formlabs, United States) according to manufacturer’s instructions unless otherwise noted. Briefly, models printed in DSG were removed from the print platform and placed in a 70% ethanol bath, gently agitated, and then incubated for 10 min. Following incubation, DSG prints were transferred to a fresh 70% ethanol bath, gently agitated, and then incubated for 10 min. Models printed in DLT were removed from the print platform and placed in a 90% ethanol bath, gently agitated, and then incubated for 2 min. Following incubation, DLT prints were transferred to a fresh 90% ethanol bath, gently agitated, and then incubated for 5 min. Following the second incubation, prints were allowed to air dry at ambient conditions. Upon drying, the lattice support structure was removed from finished models. Unless otherwise specified, models were then UV-cured using a LC3D-Print Box (Vertex-Dental B.V., Soesterberg, The Netherlands) for either 10 or 60 min.

### 2.2 Plasma treatment of 3D-printed materials

Oxygen plasma treatment was conducted on material samples using a PC 2000 Plasma Cleaner (South Bay Technology, United States). Plasma treatments were conducted under vacuum pressure of 40 Pa for 30 min with a power setting of 150 W. Samples were then used for experiments within 24 h of plasma treatment.

### 2.3 Contact angle measurements

Contact angle measurements were conducted using the sessile drop technique at ambient conditions on sample surfaces using a VCA Optima XE Video Contact Angle System (AST Products Inc., United States). For contact angle studies, a 75 mm × 16 mm x 2 mm rectangle was printed in either DSG or DLT using the approach detailed above. A 2 μL drop of distilled water was deposited on 3 different sections of each sample and the measured angles in each treatment group were averaged. For plasma treatment stability studies, printed DSG and DLT pieces were first plasma treated and then incubated at 37° for 24, 48, 72, 96 or 168 h. Contact angle measurements were then performed as described.

### 2.4 Surface oxygen content measurements

X-ray Photoelectron Spectroscopy (XPS) was performed on Dental SG and Dental LT samples using an ESCALAB 250Xi XPS (Thermo Scientific, United States) with a monochromated Al αK beam radiating at 1486.6 eV. For XPS studies, a 10 mm × 10 mm x 2 mm square was printed in either DSG or DLT using the approach detailed above. Survey scans were conducted for each sample using a 500 μm x-ray point size. Survey peaks were identified, and the percent composition of the surface was used to calculate an oxygen:carbon (O/C) ratio for each sample surface.

### 2.5 Animals

Reproductively adult female mice were obtained from a maintained breeding colony of CD-1 mice. Mice were weaned at D21 and used for experiments at 6-12 weeks of age. Upon weaning, water and Teklad Global irradiated 2916 chow containing minimal phytoestrogens and no soybean or alfalfa meal (Envigo, Madison, WI) were provided *ad libitum.* All mice were housed in a controlled barrier facility at Northwestern University’s Center for Comparative Medicine on the Chicago Campus under constant temperature, humidity, and light (14 h light/10 h dark). All animal experiments described here were approved by the Institutional Animal Care and Use Committee (Northwestern University) and were performed in accordance with National Institutes of Health Guidelines.

### 2.6 Isolation and *in vitro* maturation (IVM) of oocytes

To maximize oocyte yield, female mice were hyperstimulated with an intraperitoneal (IP) injection of 5 IU pregnant mare serum gonadotropin (PMSG, EMD Millipore, Burlington, MA), and ovaries were harvested 44–46 h post-injection. The isolated ovaries were maintained in Leibovitz’s L-15 (L15, Life Technologies Corporation, Grand Island, NY) supplemented with 3 mg/mL polyvinylpyrrolidone (PVP, Sigma-Aldrich, St. Louis, MO) and 0.5% penicillin-streptomycin (PS, Life Technologies) (L15/PVP/PS media). Milrinone, a phosphodiesterase 3A inhibitor, was added to the L15/PVP/PS media at a final concentration of 10 μM to maintain meiotic arrest (Duncan et al., 2009). Cumulus oocyte complexes were released from antral follicles by mechanical isolation as previously described (Hornick et al., 2015). Cumulus cells were mechanically removed from oocytes using a 75 μm stripper tip, and these denuded oocytes were then transferred to media consisting of α-MEM Glutamax (MEM) supplemented with 3 mg/mL bovine serum albumin (BSA) and 0.5% PS (MEM/BSA medium) with the addition of 10 μM milrinone for short-term culture in a humidified atmosphere of 5% CO_2_ in air at 37 °C. To initiate synchronous and spontaneous IVM, oocytes were rinsed through large droplets of L15/PVP/PS to remove milrinone and then transferred to appropriate vessels for IVM: polystyrene control, DSG, or DLT wells. All well sizes and volumes were the same across plates. Prior to experiments, DSG plates were autoclaved using a standard dry cycle for sterilization. DLT plates were soaked in 90% ethanol for 5 min for sterilization and then the ethanol was removed, and the plates were allowed to air dry completely under a sterile hood prior to addition of media. For each vessel, IVM was performed in a 500 μL volume of pre-equilibrated milrinone-free MEM/BSA. IVM was performed for 14–16 h at 37 °C in a humidified atmosphere of 5% CO_2_ in air. Three technical replicates of IVM were performed with between 15 and 25 oocytes/treatment groups. A total of 300 oocytes were used to study the DSG material and a total of 240 oocytes were used to study the DLT material.

To test whether the effect of DSG and DLT on meiotic maturation was due to a leachate, we generated conditioned media (MEM/BSA) that had been maintained in DLT and DSG plates for 24 h at 37 °C in a humidified atmosphere of 5% CO_2_ in air. Media kept in polystyrene plates under the same conditions served as controls. These media were then transferred into wells of polystyrene dishes (500 μL/well), and IVM was done as described above. Two technical replicates were performed with between 15 and 25 oocytes/condition for a total of 176 oocytes used in this experiment.

For IVM experiments of oocytes in the presence of a hindered amine light stabilizer, commercially known as Tinuvin 292, oocyte isolation and manipulation was performed as described above. The Tinuvin 292 mixture was resuspended in DMSO (Santa Cruz Biotechnology, Dallas, TX) at final concentrations of 0.5, 5, 50, 100, 250, and 500 mg/mL. 5 μL of each dilution was added to 4995 μL milrinone-free MEM/BSA to create the final tested concentrations. DMSO alone was used as a control. Three technical replicates of IVM were performed with between 19 and 20 oocytes/dose for a total of 459 oocytes used in this experiment.

### 2.7 Meiotic progression and chromosomal alignment analysis

Following IVM, the meiotic stage of each oocyte was scored based on established morphological criteria (Xu et al., 2009; Hirshfeld-Cytron et al., 2011). If the germinal vesicle (GV) was visible and intact, oocytes were considered arrested in prophase of meiosis I and designated as GV-intact. If the GV was absent but no polar body was observed, oocytes were designated as having undergone germinal vesicle breakdown (GVBD) and were either at pro-metaphase I or metaphase I (MI) . Cells which lacked a GV but had a polar body were considered metaphase II (MII)-arrested eggs. Oocytes that were shrunken or morphologically abnormal were classified as degenerate. Oocytes were imaged using 10X, 20X, and 40X objectives on an EVOS FL Auto live imaging microscope system (Life Technologies, Grand Island, NY, USA). MII stage eggs were fixed in 3.8% paraformaldehyde (Electron Microscopy Science, Hatfield, PA) in phosphate buffered saline (PBS) containing 0.1% TX-100 for 1 h at 37 °C. Samples were rinsed in blocking buffer (1X PBS containing 0.01% Tween-20, 0.02% NaN_3_, and 0.3% BSA) and stored at 4 °C until further processing.

To analyze spindle morphology, immunofluorescence was performed with an anti-tubulin antibody. Samples were permeabilized for 15 min under ambient conditions in 1X PBS containing 0.1% TX-100, 0.02% NaN_3_, and 0.3% BSA. Samples were then washed twice in blocking buffer and incubated in a 1:100 dilution of αTubulin (11H10) Rabbit mAb (Alexa Fluor® 488 Conjugate; Cell Signaling Technology, Danvers, MA) for 2 h at ambient conditions with gentle rocking. Following incubation, samples were washed three times in blocking buffer at ambient conditions. Samples were then mounted in Vectashield with DAPI (4′, 6-diamidino-2-phenylindole; Vector Laboratories, Burlingame, CA) and imaged using confocal microscopy. Cells were imaged on a Leica SP5 inverted laser scanning confocal microscope (Leica Microsystems) using a 63X objective and the 405 nm, 488 nm, and 543 nm lasers. For each cell, 1 μm optical thick sections were taken through the region of the spindle, and stacks or maximum projections were analyzed. Spindles that were oriented perpendicular to the image plane were excluded from the analysis. Chromosome alignment on the metaphase plate was analyzed and categorized as follows: normal (no unaligned chromosomes), 1 unaligned chromosome, >1 unaligned chromosome, and other. The “other” category includes cells that were at the incorrect cell cycle stage, as well as those that had abnormal spindle or chromosome configurations. Images were processed using LAS AF (Leica Microsystems) and Image J (National Institutes of Health, Bethesda, MD).

### 2.8 LC-MS analysis

Analysis of DLT leachate solution composition and identification of Tinuvin 292 mixture was performed using a Bruker Impact II O-TOF High Resolution Time of Flight Mass Spectrometer connected to a Bruker Elute UHPLC (Bruker Corp., Billeric, MA) in combination with Compass HyStar 4.1 Data Acquisition software for instrument operation and Compass Data Analysis for data analysis and processing. Samples were run using an ACQUITY UPLC HSS C18SB column (50 mm length, 2.1 mm ID, particle size = 1.8 μm) maintained at 40 °C. A two solvent gradient containing HPLC-grade H_2_O with 0.1% formic acid (Solvent A) and HPLC-grade acetonitrile with 0.1% formic acid (Solvent B) was used with a flow rate of 0.3 mL/min. The resulting gradient with respect to time was used for all analyses: 95% A and 5% B at 0.0 min, 95% A and 5% B at 1.0 min, 0% A and 100% B at 7.5 min, 0% A and 100% B at 9.0 min, 95% A and 5% B at 9.1 min, and 95% A and 5% B at 10.0 min. MS/MS was performed utilizing the Auto MS/MS function of the Q-TOF operating at 25 eV for collision energy.

### 2.9 Statistical analysis

All analyses were performed using statistical software, Prism 6 (GraphPad Software, La Jolla, CA). Variability within experimental groups and samples is provided as standard deviation (SD). Samples were analyzed using t-tests and one-way ANOVA. Statistical significance was determined through Welch’s correction and Tukey’s multiple comparison test.

## 3 Results

### 3.1 Production and processing of DSG and DLT culture plates

**Fig. 1:**
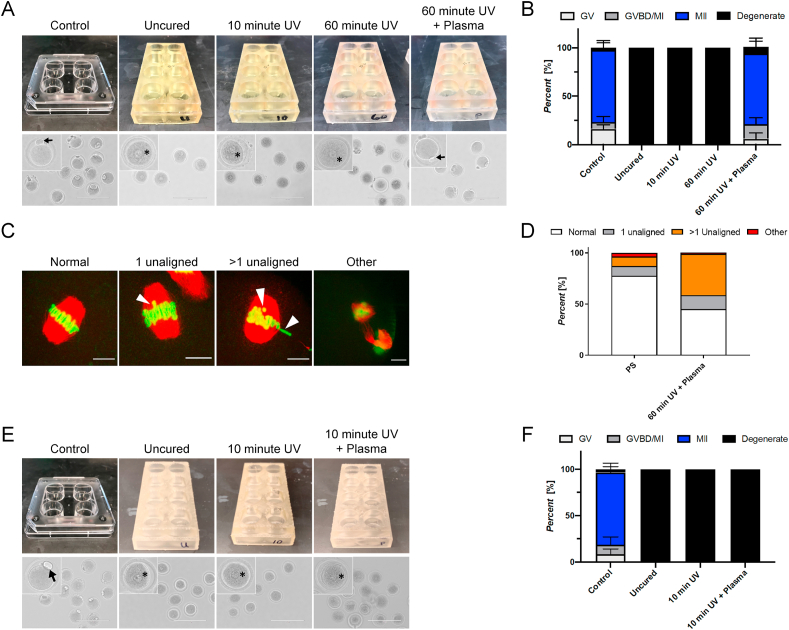
**DSG and DLT resins induce severe phenotypes in oocytesmaturedin vitro.** (A) Images of well plates used for IVM experiments and representative images of oocytes following IVM in the polystyrene control and uncured, 10-min UV cured, 60-min UV cured, and 60-min UV cured with plasma treatment DSG. Arrow in control and plasma-treated conditions highlight extruded polar body, and asterisk in other conditions highlight intact GV. Scale bar: 200 μm. (B) Resulting meiotic progression of all tested DSG conditions compared to the control. The meiotic stage of oocytes was scored as either germinal vesicle intact (GV), germinal vesicle breakdown/metaphase I (GVBD/MI), metaphase II (MII), or degenerate based on morphology. (C) Representative confocal microscopy images of chromosome and spindle architecture including normal alignment, 1 unaligned chromosome, >1 unaligned chromosome, and “other” phenotype. White arrows designate misaligned chromosomes. Scale bar: 10 μm. (D) Average incidence of chromosomal abnormalities in the control and plasma-treated DSG conditions. (E) Images of well plates and representative images of oocytes following IVM in the polystyrene control and uncured, 10-min UV cured, and 10-min UV cured with plasma treatment DLT. Arrow in control condition highlights extruded polar body and asterisk in other conditions highlight intact GV in degenerate oocytes. Scale bar: 200 μm. (F) Resulting meiotic progression incidence of all tested DLT conditions compared to the control. Error bars represent the standard deviation. Three technical replicates of IVM were performed with between 15 and 25 oocytes/treatment groups for each material. A total of 300 oocytes were used to study the DSG material and a total of 240 oocytes were used to study the DLT material.

To evaluate the effect of in vitro exposure to the two biocompatible resins, we first designed 3DP well plates containing 8 wells, with each well possessing the same dimensions as a well on a standard 24-well culture plate (diameter = 22 mm, height = 18 mm). To mimic the manner in which these materials would be used in their intended setting, we used manufacturer-recommended post-print UV curing times of 10 min for both DSG and DLT resins following ethanol washes. In addition, we characterized the effect of differing UV cure times on the biocompatibility of the materials within the context of in vitro maturation of mammalian oocytes. Additionally, as surface plasma treatment is a common method of removing impurities from material surfaces and improving the biocompatibility of plastics, such as polystyrene, the effect of oxygen plasma treatment on the biocompatibility of the materials was studied as well (Ramsey et al., 1984; Petasch et al., 1997). Complete analysis of the effects of UV-cure time and oxygen plasma treatment on the surface properties of DSG and DLT are shown in Supp. Fig. 1. Although UV curing did not affect contact angle measurements or oxygen/carbon ratios of either material, oxygen plasma treatment of both DSG and DLT resins resulted in significant changes to the surface properties of the materials, including increased wettability and corresponding oxygen:carbon (O/C) ratio (Supp. Fig. 1). The observed increase in O/C ratio is a result of the replacement of carboxyl surface groups with oxygen-containing carboxylic surface groups due to oxygen plasma treatment. This exchange of surface groups results in the increase wettability, or hydrophilicity, of material surfaces thus resulting in the significant changes on contact angle measurements (Bodas and Khan-Malek, 2007). The resulting hydrophobic-to-hydrophilic transition effect of plasma treatment on DSG and DLT surfaces was found to be relatively stable, following an initial characteristic hydrophobic recovery in the first 24 h, through one week of incubation at conditions of 37 °C and high humidity. Images of the polystyrene control plates and DSG plates are shown in Fig. 1A (DLT images appear identical and data not shown). In summary, we created custom 8-well plates, having the same well dimensions as standard culture plates, printed in both DSG and DLT resins with varied surface properties based on post-print treatment for either direct oocyte culture or for leachate isolation.

### 3.2 In vitro maturation of oocytes in DSG and DLT resins

To screen for potential reproductive toxicity of DSG and DLT resins, we used an IVM assay involving the culture of isolated murine oocytes in maturation media within the material of interest. The endpoints we used to evaluate ovo-toxicity included oocyte survival, ability to mature, and chromosome alignment on the metaphase plate. For testing of DSG resins, oocytes were matured in wells composed of the following materials: DSG untreated, 10-min UV-cured, 60-min UV cured, and 60-min UV-cured with plasma treatment. Polystyrene 4-well dishes were used as a positive control. Following 16 h of IVM, the majority (75.9% ± 9.3%) of cells in the control group matured to metaphase of meiosis II (MII) as characterized by the extrusion of a polar body as expected (Fig. 1A). However, IVM performed in DSG plates that were not plasma-treated resulted in complete degeneration of all oocytes (Fig. 1A and B). One of the first events that occurs during spontaneous meiotic maturation is the breakdown of the nuclear envelope of the germinal vesicle (GV), which typically occurs 1–3 h after initiation of IVM. Interestingly, even though oocytes that were matured in non-plasma treated DSG materials were all degenerate, they appeared to contain intact germinal vesicles (Fig. 1B). This observation suggests that degeneration occurred rapidly upon exposure to the material and prior to resumption of meiosis. DSG well plates that were plasma-treated prior to IVM experiments had a less severe effect as meiotic progression of oocytes matured under these conditions was comparable to controls, with 75.3 ± 10.5% oocytes matured in plasma-treated DSG reaching the MII stage compared to 74.3 ± 9.9% in the control. Meiotic progression rates in the control and plasma-treated conditions are further detailed in Supp. Fig. 2. These results indicate that exposure of oocytes to DSG resin induces rapid degeneration of gametes; however, plasma treatment of the material mitigates oocyte degeneration.

Upon completion of meiotic maturation, oocytes arrest at metaphase of meiosis II and are characterized by a bipolar spindle with chromosomes aligned on the metaphase plate (Fig. 1C). Disruption of this phenotype, including misalignment of chromosomes or abnormal spindle morphology, can result in reduced oocyte quality and aneuploidy (Jones and Lane, 2013). Although plasma treatment appeared to confer a protective effect on the oocytes, resulting in MII-arrested eggs, we wanted to further assess chromosomal alignment as an additional determinant of gamete quality. Therefore, we assessed spindle morphology using immunofluorescence. Following IVM in plasma-treated DSG, only 43.0 ± 37.16% of MII stage eggs exhibited normal chromosome alignment relative to 80.6 ± 17.3% in the control. Further, while the percent of single-chromosome misalignment were similar for both plasma-treated DSG and the control conditions (11.9 ± 2.7% and 11.1 ± 19.2%, respectively), the incidence of spindles that exhibited a more severe phenotype (e.g. multiple chromosomes misaligned on the metaphase plate) was 9.29 ± 11.7% in the MII eggs of the control group compared to 42.0 ± 36.4% of the MII eggs exposed to plasma-treated DSG (Fig. 1D, Supp. Fig. 3). These results demonstrate that while plasma-treatment of DSG prevented oocyte degeneration in culture, DSG resin nevertheless resulted in abnormal gametes with an increased incidence of chromosome misalignment.

Similar to DSG resins, oocytes were matured in vitro within DLT resins in the following experimental cohorts: untreated, 10-min UV-cured, and 10-min UV-cured with plasma treatment plates. DLT material UV-cured for time periods longer than 10 min exhibited structural deformation, and therefore curing times longer than 10 min were not tested. Oocytes matured in DLT plates degenerated in all conditions, including plasma-treated DLT (Fig. 1E and F). Similar to the DSG conditions without plasma treatment, the degenerate oocytes appeared to still have an intact GV, indicating that rapid degeneration occurred prior to the resumption of meiosis. Because all oocytes degenerated and failed to reach the MII stage following exposure to DLT under all conditions, further analysis of spindle morphology and chromosome alignment was not possible.

### 3.3 Time course analysis of in vitro maturation kinetics in DSG and DLT

**Fig. 2:**
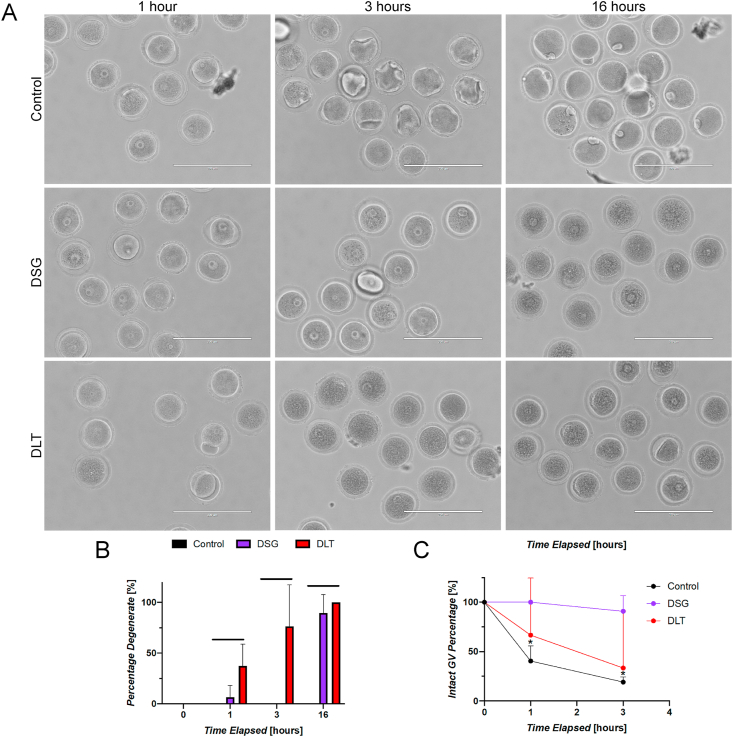
**Time course analysis of meiotic progression and oocyte degeneration in DSG and DLT resins.** (A) Representative images of oocytes after 1, 3, and 16 h during the course of IVM within the polystyrene control, untreated DSG, and untreated DLT resins. (B) Percentage of degenerate oocytes cultured within each material at 0, 1, 3, and 16 h showing rapid degeneration of oocytes matured in DLT resins. (C) Percentage of oocytes with intact germinal vesicles at 0, 1, and 3 h of culture in each material showing altered kinetics of meiotic progression evidenced by a greater percentage of cells arrested in the GV stage in oocytes matured in the DSG resin. Scale bar: 200 μm. Error bars represent the standard deviation. Statistical significance was determined by (B) one-way ANOVA and (C) Tukey’s multiple comparison test (∗*p* < 0.05, ∗∗*p* < 0.01, ∗∗∗∗*p* < 0.0001, ns = not significant). Three technical replicates of time course analysis were performed with between 11 and 21 oocytes/material per time point for a total of 408 oocytes used for this experiment.

To further understand the time course and the underlying mechanisms of the induced toxicity due to incubation in DSG and DLT resins, we compared the kinetics of meiotic maturation in oocytes cultured in either polystyrene, DSG, or DLT plates at 1, 3, and 16 h post-initiation of IVM (Fig. 2A). At 1-h post initiation of IVM, all oocytes were morphologically normal in the control condition and very few degenerate oocytes were observed in the DSG condition (6.7 ± 11.5%); however, a significant number of oocytes within the DLT resin (37.4 ± 21.3%) were degenerate at this time-point (Fig. 2B). Quantification of GV stage oocytes after 1 h of culture revealed a decrease in the percentage of intact GVs within the control condition (40.5 ± 15.4%) as oocytes transitioned to the GVBD/MI stage. There was also a decrease in GV oocytes within the DLT condition (66.7 ± 57.7%) that correlated with the percentage of degenerate oocytes (Fig. 2C). Interestingly, all oocytes cultured within DSG resin remained at the initial GV stage at the same time point, indicating a delay in the onset of meiotic maturation. At the 3-h time point, while no oocytes in either the control or DSG groups were degenerate, the percentage of degenerate oocytes within the DLT condition increased to 76.5 ± 40.8%. Further, the percentage of GV stage oocytes within the DLT condition continued to decrease proportional to the percentage of degenerate oocytes after 3 h (33.3 ± 57.7%). Within the control condition, the percentage of GV stage oocytes was also found to be lower at the 3-h time-point (19.2 ± 5.1%) as oocytes continued to mature to the GVBD/MI stage. Conversely, oocytes within the DSG condition remained largely at the GV stage (90.9 ± 15.7%) at 3 h following initiation of IVM. These results demonstrate that while DLT causes degeneration, DSG causes a significant delay in meiotic resumption. After 16 h, all oocytes matured in untreated DLT were degenerate consistent with what was observed in previous experiments (Fig. 1F). Similar to DLT, overnight incubation in DSG resins resulted in degeneration of the majority of the oocytes (89.7 ± 17.7%), compared to no degeneration in controls. These results demonstrate that DSG exposure caused oocytes to remain arrested in prophase I during the first 3 h of IVM through an unknown mechanism, and this was followed by degeneration following 16 h of culture. Exposure to DLT, however, induced rapid degeneration of oocytes within the first few hours of culture indicating that DLT exposure is more toxic to oocytes compared to DSG.

To determine whether the effects of DSG and DLT were due to direct contact with the material, we performed IVM using conditioned media that had been pre-incubated in DSG and DLT plates. Whereas 92.23 ± 6.93% of the cells matured in media pre-incubated in control polystyrene dishes reached the MII stage, meiotic progression was significantly compromised following IVM in media pre-incubated in DSG and DLT plates, with 39.52 ± 47.51% and none of the oocytes reaching the MII stage, respectively (Supp. Fig. 4). These results provide further evidence that DLT causes a more severe phenotype than DSG and demonstrate that conditioned media from these resins alone is capable to cause meiotic phenotypes which partially recapitulate what are observed when oocytes are matured in plates made of these materials. Thus, we performed mass spectroscopy analysis of conditioned media to identify any observable changes to media composition following incubation within DLT.

### 3.4 Mass spectroscopy analysis of DLT leachate

**Fig. 3:**
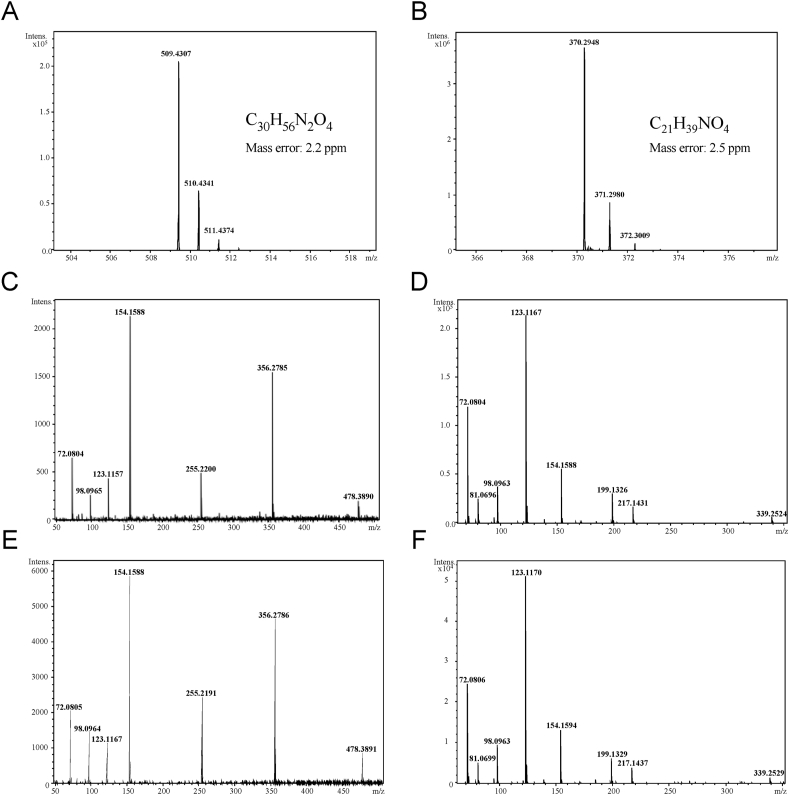
**Mass-spec analysis of DLT leachate and identification of Tinuvin 292 mixture.** Zoom-in raw MS spectra for dominant peaks identified with elution times of (A) 4.8–5.0 min and (B) 0.9–1.0 min from the DLT leachate. Tandem mass spectroscopy (MS/MS) was performed to create fragmentation spectra for the (C) 370 and (D) 509 ions. MS/MS of similar ions identified in Tinuvin 292 result in similar MS/MS spectra for the (E) methyl 1,2,2,6,6-pentamethyl-4-piperidyl sebacate and (F) bis(1,2,2,6,6-pentamethyl-4-piperidyl) sebacate components of the mixture. For display purposes, the parent mass was ignored in spectra creation and the acquisition low mass cutoff was used for the low mass limit and the highest fragment mass created via MS/MS was used for the high mass limit displayed in spectra to show detail of the relevant product masses.

Due to the severity of degeneration of oocytes exposed to the DLT resin and our inability to rescue the effects with plasma treatment as seen with the DSG resin, we set out to identify any compounds that may be released from printed DLT responsible for the observed negative phenotypes. The remaining studies were therefore performed using DLT. To identify potential reproductive toxins that are released from the DLT resin, media was incubated as before within 8-well plates that were printed in DLT and processed according to the manufacturer’s instructions. Media was also incubated within polystyrene 4-well plates as a control. Media samples were then analyzed using LC-Q-sTOF mass spectrometry and the DLT media was compared to the control media to identify any differing peaks. Two prominent peaks were identified at elution times of 0.9–1.0 and 4.8–5.0 min that were unique to media incubated within DLT wells. The raw MS spectra show ion peaks of *m*/*z* = 370 and *m*/*z* = 509 in Fig. 3A and B, respectively. Based on the components known to be contained within the DLT resin according the material’s SDS, as shown in Table 1, compound formulas of C_21_H_39_NO_4_ and C_36_H_56_N_2_O_4_ were determined to be the most likely source of the observed peaks. Fragmentation of the *m*/*z* = 370 and *m*/*z* 509 ions via tandem mass spectroscopy (MS/MS) resulted in the fragmentation spectra shown in Fig. 3C and D, respectively. Analysis of these fragmentation spectra in combination with the proposed chemical formulas utilizing the MetFrag database resulted in the identification of potential structures, a majority of which were found to be variants of either methyl 1,2,2,6,6-pentamethyl-4-piperidyl sebacate or bis(1,2,2,6,6-pentamethyl-4-piperidyl) sebecate, the pentamethyl-piperidyl sebacate (EG No. 255-437-1) specified in the DLT SDS provided by the manufacturer. These two compounds are commonly sold commercially as Tinuvin 292, a mixture of bis- and methyl 1,2,2,6,6-pentamethyl-4-piperidyl sebacate in a 3:1 ratio. MS/MS fragmentation of the peaks observed Tinuvin 292 mixture resulted in the spectra, shown in Fig. 3E and F, which show exact fingerprint matches when compared to the fragmentation spectra of the *m*/*z* = 370 and *m*/*z* = 509 ions observed within the DLT leachate. Furthermore, the MS/MS fragmentation patterns are readily rationalizable fragments from the parent ion structure, further supporting identification and standard match of Tinuvin 292 (see supporting information in Supp. Fig. 6). Following confirmation of the presence of both components of Tinuvin 292 within the DLT leachate, mass spectroscopy was utilized to determine the concentration of Tinuvin 292 within the media following 16 h of incubation to be approximately 50 μg/mL. These results indicate that the components of the Tinuvin 292 mixture, a commercially available hindered amine light stabilizer, are released from the DLT resin even after following the manufacturer’s protocol for ensuring biocompatibility.

**Table 1:** Hazardous ingredients disclosed in Dental SG and Dental LT Clear SDS.

Resin Type	Hazardous Ingredients	Approximate % w/w
Dental SG (DSG)		
	Methacrylic oligomers	>90
	Phosphine oxides	<3
Dental LT Clear (DLT)		
	Methacrylic oligomer	>70
	Glycol methacrylate	<20
	Pentamethyl-piperidyl sebacate	<5
	Phosphine oxide	<2.5

### 3.5 **Exposure of oocytes to Tinuvin 292 alone recapitulates the meiotic phenotypes induced by DLT**

**Fig. 4:**
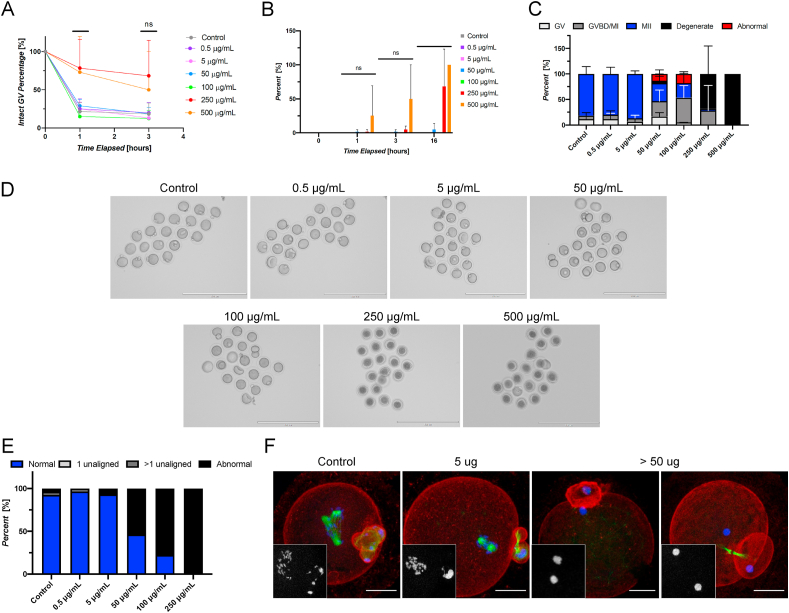
**Tinuvin 292 induces abnormal meiotic phenotypes and oocyte degeneration in a dose-responsive manner in vitro.** (A) Percentage of oocytes with intact germinal vesicles at 0, 1, and 3 h of culture in each material showing altered meiotic kinetics as evidenced by increased percentage of the GV-intact phenotype in oocytes exposed to concentrations of 250 and 500 μg/mL. (B) Percentage of oocytes determined to be degenerate at 0, 1, 3, and 16 h showing rapid degeneration of oocytes matured in the presence of 250 and 500 μg/mL (C) Resulting meiotic progression rates of oocytes matured in the presence of Tinuvin 292 showing meiotic disruption at concentrations of ≥50 μg/mL. (D) Representative images of oocytes following IVM in the presence of a range of concentrations of Tinuvin 292. Scale bar: 200 μm. *(*E) Average incidence rates of chromosomal abnormalities showing dose response effect of Tinuvin 292. (F) Representative confocal microscopy images of abnormal phenotypes observed in control, 5 μg/mL, and the 50, 100, and 250 μg/mL conditions. Insets show DNA. Scale bar: 25 μm. (Error bars represent the standard deviation. Statistical significance determined using a one-way ANOVA (∗p < 0.05, ∗∗p < 0.01, ∗∗∗∗p < 0.0001, ns = not significant). Three technical replicates of IVM were performed with between 19 and 20 oocytes/dose for a total of 459 oocytes used in this experiment.

To determine whether Tinuvin 292 was alone responsible for the phenotypes observed following incubation of oocytes within DLT wells, we performed IVM of oocytes in media containing a dose response series of Tinuvin 292 ranging from 0 to 500 μg/mL. Following 1-h of culture, exposure to Tinuvin 292 concentrations of up to 100 μg/mL did not appear to affect the ability of oocytes to resume meiosis (Fig. 4A). However, there was a clear effect on meiotic resumption at concentrations of 250 μg/mL and 500 μg/mL where 78.3 ± 37.5% and 73.0 ± 46.7% of cells maintained intact GVs at 1-h, respectively. This is in contrast to controls in which the majority of the oocytes had undergone GVBD, and only 21.7 ± 11.5% remained GV intact at the 1-h time point (Fig. 4B). At the conclusion of the culture period, a majority of oocytes (68.3% ± 54.8%) were degenerate in the 250 μg/mL condition, and all oocytes were degenerate at 500 μg/mL. Further assessment of meiotic progression revealed meiotic disruption at concentrations as low as 50 μg/mL (Fig. 4C and D, Supp. Fig. 5). Indeed, our studies show a dose dependent inhibition of meiotic progression and a high incidence of oocyte degeneration.

Analysis of chromosomal alignment of MII stage eggs in each treatment group revealed a dose-response effect of Tinuvin 292, as the incidence rate of abnormal phenotypes increased with increasing concentrations (Fig. 4E). Taken together, these experiments demonstrate that Tinuvin 292, a commercial hindered amine light stabilizer mixture, induces severe reproductive toxicity in vitro, including meiotic disruption, abnormal phenotypes, and gamete degeneration at concentrations as low as 50 μg/mL.

## 4 Discussion

Although both DSG and DLT are marketed as “biocompatible” photopolymers, our results demonstrate that the context for biocompatibility is critical. When processed according to the manufacturer’s protocols, exposure to either DSG or DLT induced rapid mammalian oocyte degeneration in vitro. Plasma treatment of DSG, but not DLT, was an effective method for preventing oocyte degeneration; however, a significant increase in chromosomal abnormalities was still observed. Based on the results of time course experiments, the negative outcomes induced by DSG and DLT differ in timing and intensity, with exposure to the DLT leachates inducing a more severe phenotype that resulted in rapid gamete degeneration prior to the resumption of meiosis. We focused on identifying the leachate from DLT since this was determined to be the more toxic biomaterial and elicited the most severe phenotypes in oocytes which were not mitigated through plasma treatment. We also chose to focus on DLT because this material is intended for use in situations in which individuals would be exposed longitudinally for extended periods of time. Whereas DSG is intended for use in manufacturing dental surgical guides which would likely be used in a patient on the scale of minutes, DLT is intended for manufacturing oral retainers, which would likely be worn for hours at a time across an extended period. In so doing, we identified the release of methyl 1,2,2,6,6-pentamethyl-4-piperidyl sebacate and bis(1,2,2,6,6-pentamethyl-4-piperidyl) sebacate, a mixture commercially known as Tinuvin 292, from the DLT material as confirmed by mass spectroscopy and dose response experiments. Importantly, our observations that Tinuvin 292 has direct toxicity effects on isolated oocytes suggest that it may function differently than a typical endocrine disruptor. We did not isolate the leachate from DSG, but we suspect the active factor may not be Tinuvin 292 since the effects on the oocyte differ between the resins. Although beyond the scope of the current study, it will be important in the future to determine what residual leachates are still present in plasma-treated DSG resin that are causing meiotic errors despite the ability of cells to still progress through meiosis.

A challenge to using photocurable resins, such as SLA photopolymers, is the need for inclusion of photoinitiators and light stabilizers that may be released from the material if not sufficiently reacted or washed away during post-print processing. One of the most common classes of light stabilizers are hindered amine light stabilizers (HALS). HALS are used in the production of a large number of plastics, including polyethylene, polystyrene, polypropylene, and polycarbonate, and are often used in coatings to prevent oxidation damage due to their ability to repeatedly react with radicals that are formed during photo-oxidative degradation (Hodgson and Coote, 2010). This also makes HALS excellent for the prevention of radical polymerization in photopolymers. However, while the source of the release of these compounds was a photopolymer, our findings have widespread significance as the identified compound is part of a class of polymer additives that are widely used in both consumer and medical products (Zhang et al., 1986; Glossmann et al., 1993; Papke et al., 1994; Marqué et al., 1996; Klampfl and Himmelsbach, 2016). These compounds readily leach from plastic toys and baby bibs into saliva, raising substantial concerns with regards to their use in dental structures printed in DLT (Noguerol-Cal et al., 2011; Rajbux et al., 2020). As such, exposure to these additives due to their release from polymers over time must be considered with regards to their use in plastic manufacturing.

Although the results of this study are concerning with regard to the exposure of oocytes to either DSG or DLT resins, it is difficult to extrapolate our findings to the potential in vivo toxicity of these resins. Further examination of systemic and targeted effects resulting from in vivo exposure of either the resins or their leachates are warranted. However, whole animal models that accurately replicate the use-case of DSG and DLT and the resulting exposure are not trivial. Additionally, it will also be important to investigate the reproductive toxicity on the male reproductive system both in vitro and in vivo to identify any sex differences in the response to exposure to DSG, DLT, and their leachates. Lastly, specific characterization of the release and the resulting exposure levels of Tinuvin 292 in humans, both in the case of medical products such as those made using DLT as well as consumer products, should be further studied given our findings of severe reproductive toxicity following leaching from plastic materials.

Despite the importance of reproductive health, certification of biocompatibility by the International Organization for Standardization (ISO) does not require reproductive health safety testing, except in cases where material come into direct contact with reproductive tissues (Standardization, 2014). BPA represents one of the most well-known plastic additives initially used in commercial products that was later shown to induce cytotoxic effects. It was first identified as a leachate from polycarbonate during the process of autoclaving in a lab that was studying gamete biology. While initial studies found that BPA exposure induces deleterious meiotic effects in the oocyte, additional studies have shown a much broader capacity to cause adverse outcomes across reproductive systems in both animal models and humans (Krishnan et al., 1993; Hunt et al., 2003; vom Saal et al., 2007). Thus, the initial identification of toxicity within the oocyte was the linchpin discovery that ultimately resulted in the removal of BPA from most consumer products (Hunt et al., 2009; Brieno-Enriquez et al., 2011; Gorence et al., 2019; Vandenberg et al., 2019). Beyond BPA, there have been a large number of studies investigating the impact of plasticizers and other plastic additives that can act as endocrine disrupting chemicals and negatively impact reproductive health and these studies have been thoroughly reviewed elsewhere (Brehm and Flaws, 2019, Crain et al., 2008, Ghisari and Bonefeld-Jorgensen, 2009, Giulivo et al., 2016, Latini et al., 2004, Lyche et al., 2009, Meeker et al., 2009, Segal and Giudice, 2019).

The work in this paper represents the first demonstration of the reproductive toxicity of DSG or DLT resins/leachates in vitro. Although no toxicity data are provided by the manufacturer, there are two previous studies in which the potential toxicity of DSG was investigated. In one study, a zebrafish embryo culture model was used to characterize effects of exposure to DSG resins “as-built” (i.e. no ethanol wash and no post-print UV cure), UV-cured but not ethanol washed, and UV-cured and ethanol washed (Alifui-Segbaya et al., 2018). Although negative outcomes, including lethality and developmental defects, were observed in the first two conditions, no negative effects were observed in ethanol-washed and UV-cured DSG (the equivalent of the 10-min UV cured DSG in this study). A second study evaluated DSG exposure in a murine fibroblast (L929) cell line and gingival fibroblasts and found an overall decrease in metabolic activity in both cell types (Kurzmann et al., 2017). No toxicity studies concerning DLT are in the literature to date. These previous studies, as well as the results reported herein, indicate that the certification of biocompatibility of DSG and DLT should be contextualized and further evaluated. The need for more stringent testing of potential reproductive toxicity of new 3DP materials will likely continue to grow as additive manufacturing technologies have become increasingly desirable for medical applications where rapid manufacturing of patient-specific tools is required, such as the case with aligners for treatment of dental malocclusions. While resins such as DLT allow the manufacturing of products that more readily address misalignments compared to other approaches such as orthodontic brackets, as our results show it may also expose patients to compounds that leach from the material and could negatively impact reproductive health.

## 5 Conclusions

In summary, the use of two 3DP resins within a reproductive biology laboratory unexpectedly revealed severe reproductive toxicity following both direct and indirect exposure of murine oocytes to these materials in vitro, despite their ISO-certification of biocompatibility and commercial marketing for use in dental applications. These results illustrate the ability of the oocyte to be a sensitive and effective cell type for the study of reproductive effects of new materials. Additionally, the identification of the release of Tinuvin 292 from the DLT resin and confirmation of its ability to induce detrimental effects on the oocyte highlight the need for clarification of biocompatibility certification of materials and suggest that the potential of Tinuvin 292, as well as similar compounds, to induce similar effects in vivo should be further investigated.

## Author Credit statement

**Hunter B. Rogers:** Investigation, Data curation, Writing- Original draft preparation, Visualization. **Luhan T. Zhou:** Data curation, Visualization, Software, Writing- Reviewing and Editing. **Atsuko Kusuhara:** Data curation. **Emily Zaniker:** Data curation. **Saman Shafaie**: Data curation. **Benjamin C. Owen:** Data curation. **Teresa K. Woodruff:** Supervision, Validation, Writing- Reviewing and Editing. **Francesca E. Duncan:** Supervision, Validation, Writing- Reviewing and Editing.

## Declaration of competing interest

The authors declare that they have no known competing financial interests or personal relationships that could have appeared to influence the work reported in this paper.
