# Supplementary material for: Dental resins used in 3D printing technologies release ovo-toxic leachates
Source: Chemosphere. 2021 May;270:129003. doi: 10.1016/j.chemosphere.2020.129003 (PMC7957323; doi:10.1016/j.chemosphere.2020.129003)
Supplement: Multimedia component 1 [file mmc1.docx]

**Supplementary Figure Legends**

**Supplemental Figure 1: Characterization of Surface Properties of DSG and DLT resins following UV-curing and plasma treatment.** (A) Representative images from contact angle measurements of uncured DSG, 60-minute UV-cured plasma-treaded DSG, uncured DLT, and 10 minute UV-cured and plasma-treated DLT. (B) Average contact angle measurements of DSG resin following UV-curing and plasma treatment (N = 3 for each condition). (C) XPS analysis of O/C surface content of DSG resin following UV-curing and plasma treatment (N = 3). (D) Contact angle measurements of untreated and 60-minute UV-cured and plasma-treated DSG at t = 0, 24, 48, 72, and 168 hours of incubation. (E) Average contact angle measurements of DLT resin following UV-curing and plasma treatment (N = 3 for each condition). (F) XPS analysis of O/C surface content of DLT resin following UV-curing and plasma treatment (N = 3). (G) Contact angle measurements of untreated and 10-minute UV-cured and plasma-treated DSG at t = 0, 24, 48, 72, and 168 hours of incubation. Error bar represent the standard deviation. Statistical significance determined using two-tailed t-test with comparison to the uncured material (* p < 0.05, ** p < 0.01, *** p < 0.0001, ns = not significant).

**Supplemental Figure 2. Statistical analysis of DSG meiotic progression incidence.** Average percentages of (A) GV stage, (B) GVBD/MI stage, (C) MII stage, and (D) degenerate oocytes following *in vitro* maturation in polystyrene and DSG wells. Error bars represent the standard deviation. Statistical significance in (A-C) determined using unpaired t-test with Welch’s correction (* p < 0.05, ns = not significant). Statistical significance in (D) determined using Tukey’s multiple comparison test.

**Supplemental Figure 3. Statistical analysis of DSG spindle/chromosomal abnormality incidence.** Average percentages of (A) normal spindle morphology, (B) single chromosome misalignment, (C) multiple (>1) chromosome misalignment, and (D) undefined abnormal phenotypes in MII oocytes following *in vitro* maturation in polystyrene and DSG wells. Error bars represent the standard deviation. Statistical significance determined using unpaired t-test with Welch’s correction (ns = not significant).

**Supplemental Figure 4. Leachate from DSG and DLT resins are sufficient to cause meiotic defects.** Oocytes were matured in media that had been conditioned for 24 hours in polystyrene (PS), DSG, and DLT. (A) The percentage of cells in each meiotic stage were scored following IVM. Cells were considered either germinal vesicle intact (GV), germinal vesicle breakdown/metaphase I (GVBD/MI), metaphase II (MII), or degenerate based on observed morphology. (B) Average incidence of chromosomal abnormalities in MII stage eggs following IVM in conditioned media from PS and DSG plates. We could not obtain this information for DLT because no MII stage eggs were obtained in this experimental cohort. Statistical significance was determined by (A) Two-way ANOVA (P=0.0002) and (B) Two-way ANOVA (ns).

**Supplemental Figure 5. Statistical analysis of meiotic progression incidence following exposure to Tinuvin 292**. Average percentages of (A) GV stage, (B) GVBD/MI stage, (C) MII stage, (D) degenerate, and (E) abnormal phenotype oocytes following *in vitro* maturation in different concentration of Tinuvin 292. Error bars represent the standard deviation. Statistical significance determined using Tukey’s multiple comparison test.

**Supplemental Figure 6. Proposed structures for the neutral losses observed in the MS/MS spectra for ions of m/z 370 and ions of m/z 509.** The structural analysis of these fragmentation patterns for (A) C_30_H_57_N_2_O_4_^+^ and (B) C_21_H_40_NO_4_^+^ further confirms the unknown compound identity as Tinuvin 292 light mixture. Additionally, mass spectral abundance difference for ions of 154 and 123 (more 154 for the bis(1,2,2,6,6-pentamethyl-4-piperidyl) compound due to having two points of substitution) and the 199 fragment ion being produced from the ion of m/z 370 only (demonstrating the methyl substituted sebacate, in this case) both confirm the structural differences between the compounds and their presence in the leachate due to this unique fragmentation pattern.
