# Supplementary material for: Dental resins used in 3D printing technologies release ovo-toxic leachates
Source: Chemosphere. 2021 May;270:129003. doi: 10.1016/j.chemosphere.2020.129003 (PMC7957323; doi:10.1016/j.chemosphere.2020.129003)
Supplement: Multimedia component 2 [file mmc2.pptx]

## Slide 1
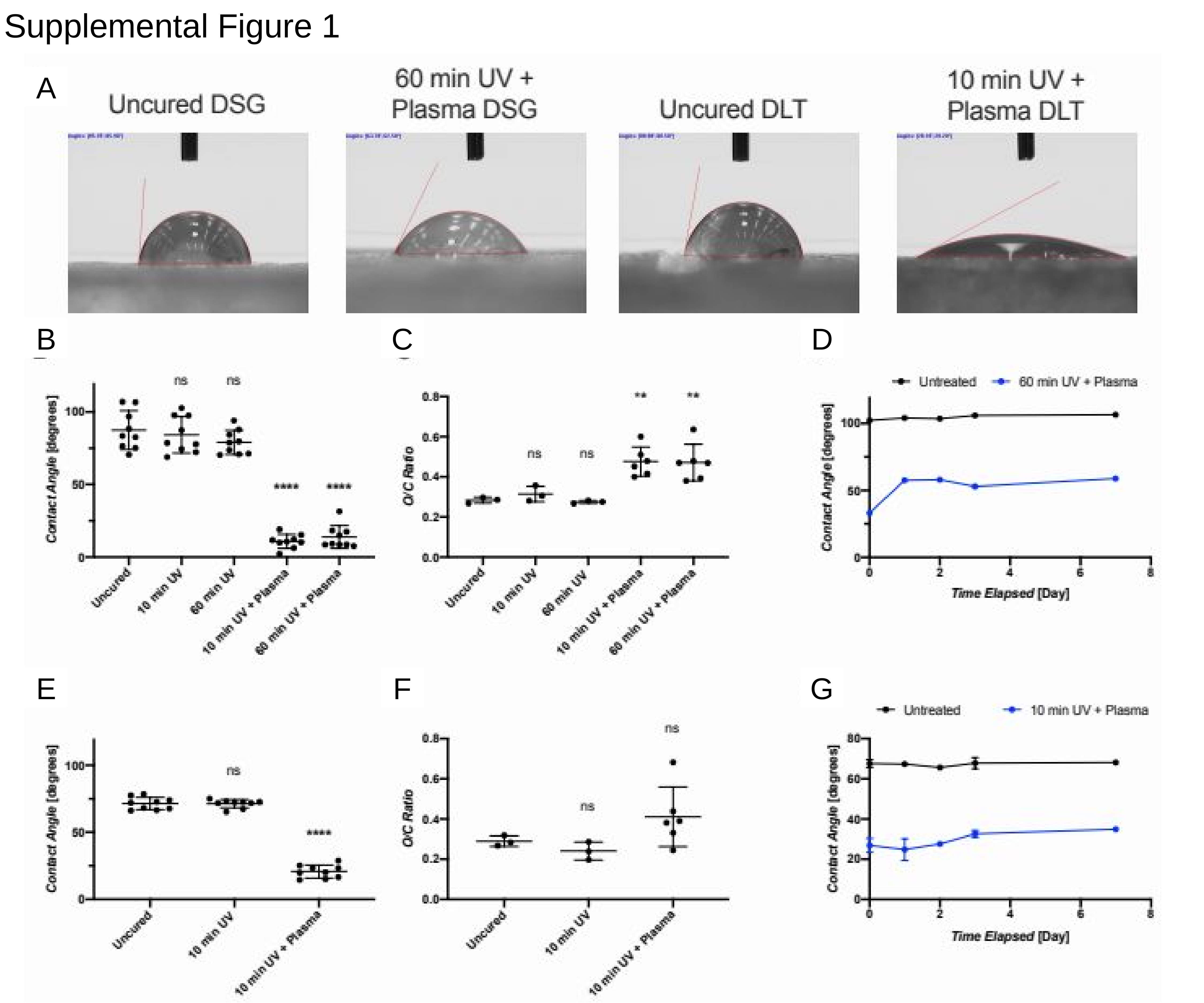

# Supplemental Figure 1
A
B
C
D
E
F
G

## Slide 2
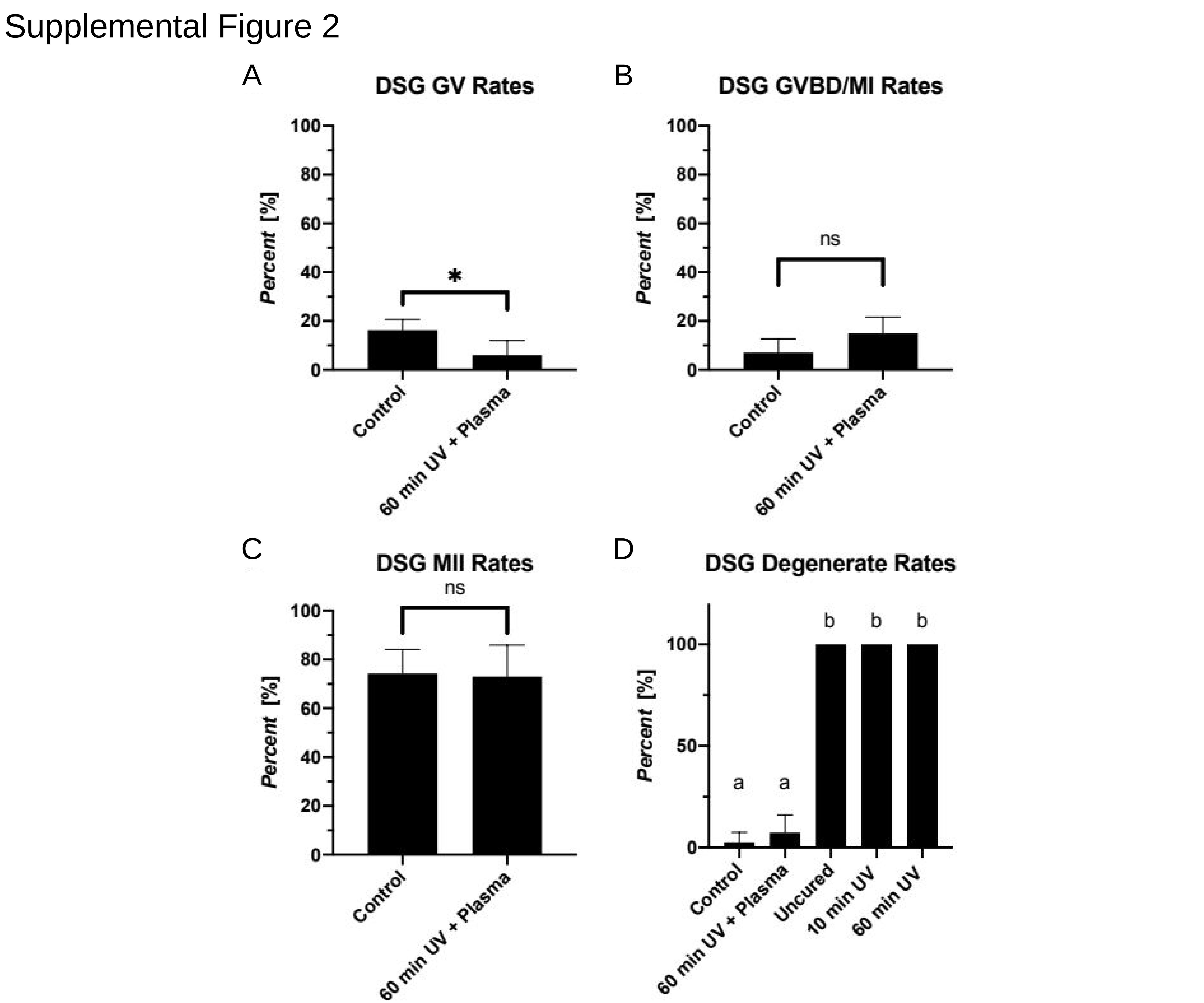

# Supplemental Figure 2
A
B
C
D

## Slide 3
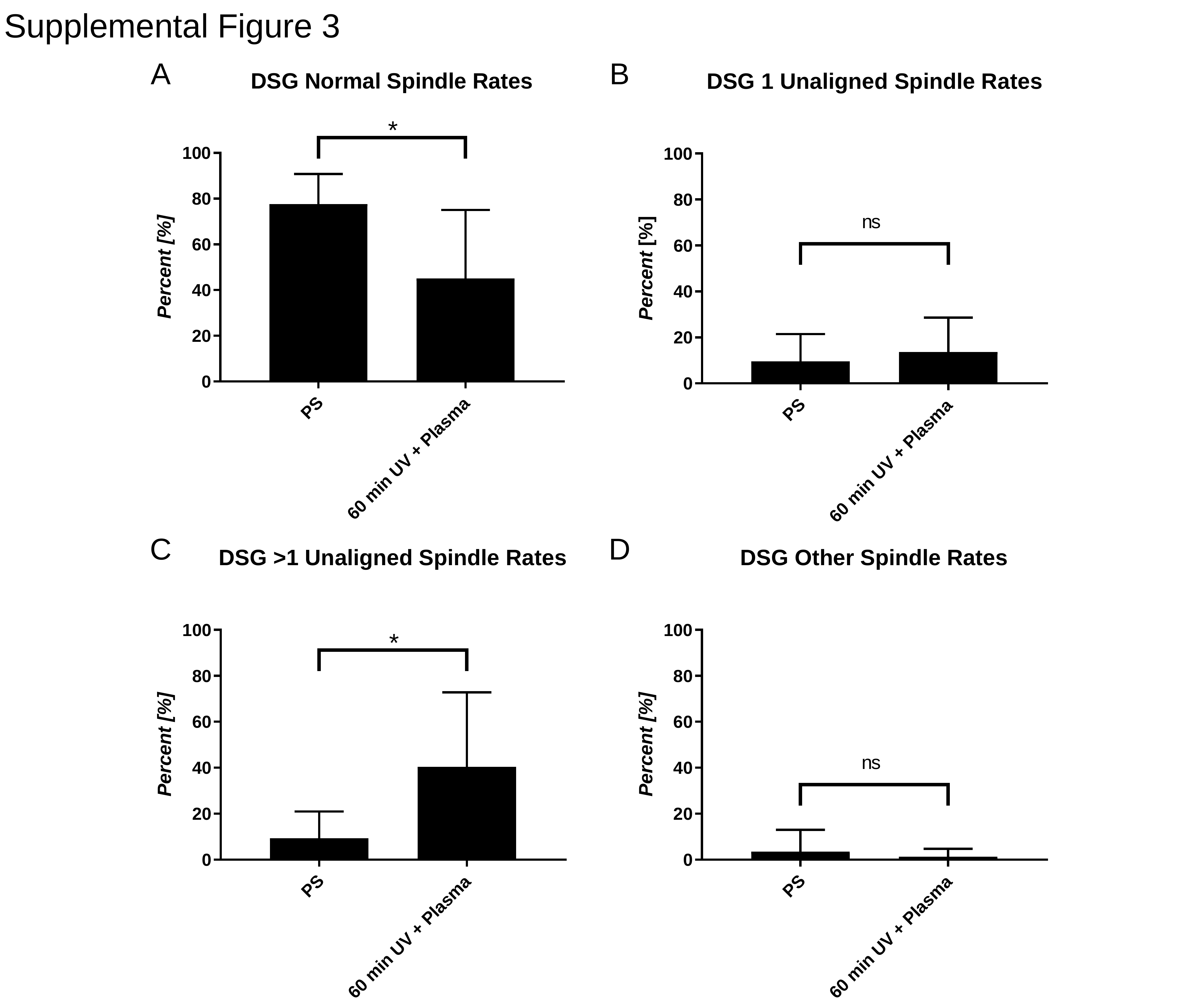

# Supplemental Figure 3
A
B
C
D

## Slide 4
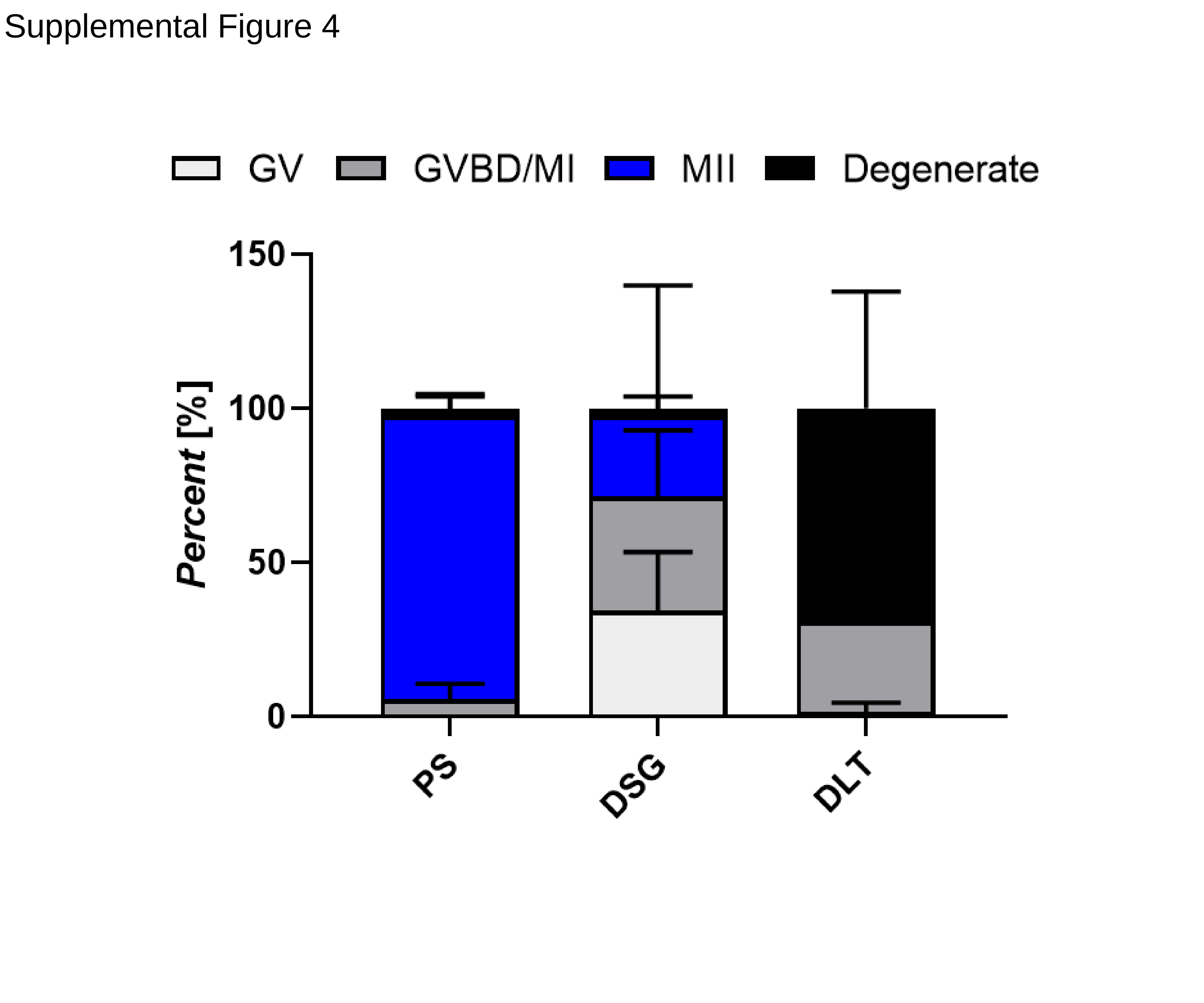

# Supplemental Figure 4

## Slide 5
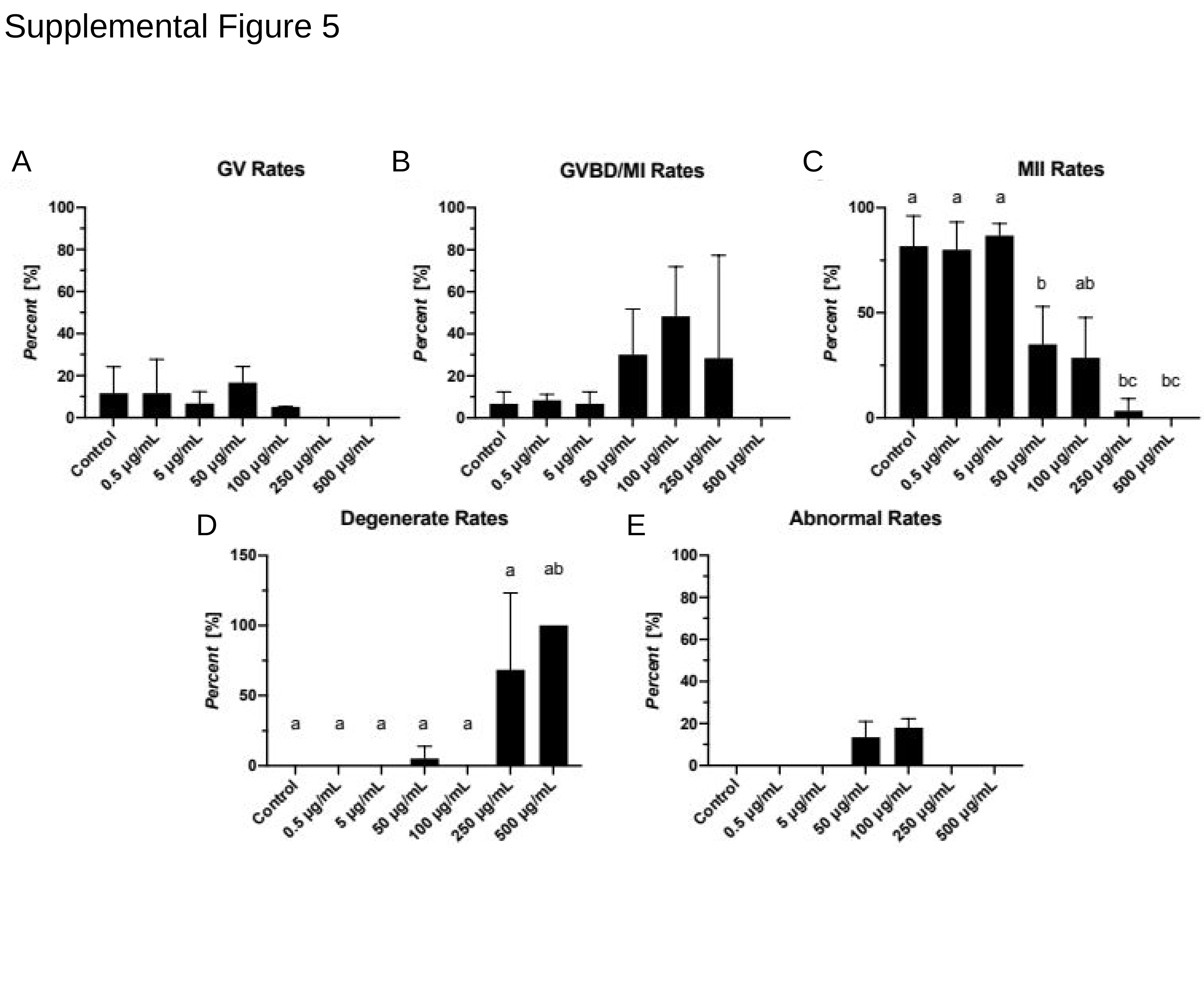

# Supplemental Figure 5
A
B
C
D
E

## Slide 6
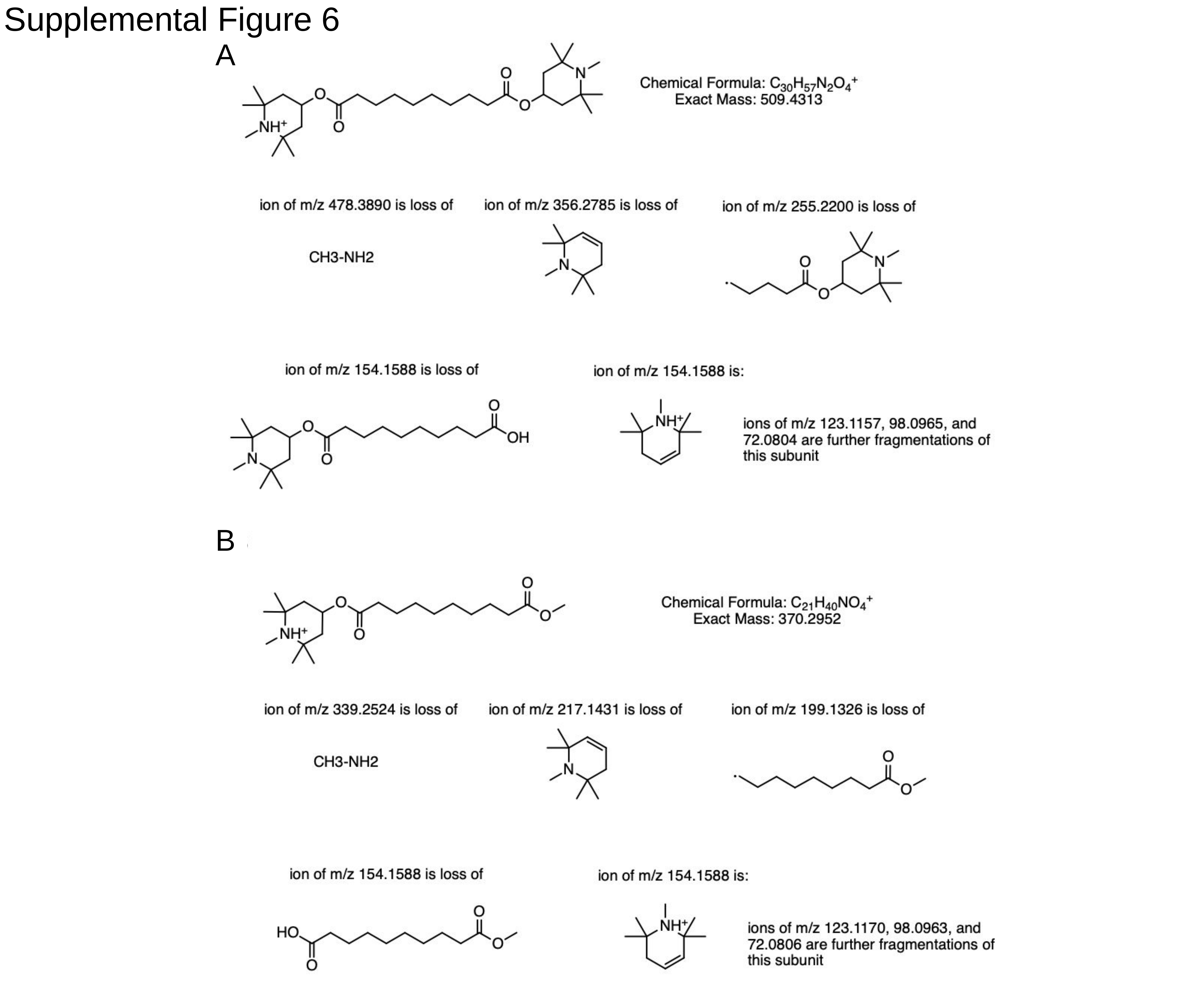

Supplemental Figure 6
A
B
